# SARS-CoV-2 European resurgence foretold: interplay of introductions and persistence by leveraging genomic and mobility data

**DOI:** 10.21203/rs.3.rs-208849/v1

**Published:** 2021-02-10

**Authors:** Philippe Lemey, Nick Ruktanonchai, Samuel L. Hong, Vittoria Colizza, Chiara Poletto, Frederik Van den Broeck, Mandev S. Gill, Xiang Ji, Anthony Levasseur, Adam Sadilek, Shengjie Lai, Andrew J. Tatem, Guy Baele, Marc A. Suchard, Simon Dellicour

**Affiliations:** 1 Department of Microbiology, Immunology and Transplantation, Rega Institute, KU Leuven, Leuven, Belgium; 2 Global Virus Network (GVN), Baltimore, MD, USA; 3 WorldPop, School of Geography and Environmental Science, University of Southampton, Southampton SO17 1BJ, UK; 4 Population Health Sciences, Virginia Tech, Blacksburg, VA, USA; 5 INSERM, Sorbonne Université, Institut Pierre Louis d’Epidémiologie et de Santé Publique IPLESP, F75012 Paris, France; 6 Department of Biomedical Sciences, Institute of Tropical Medicine, Antwerp, Belgium; 7 Department of Mathematics, School of Science & Engineering, Tulane University, New Orleans, LA, USA; 8 Microbes, Evolution, Phylogeny and Infection, Aix-Marseille Université and Marseille Institut Universitaire de France, Marseille, France; 9 Google, Mountain View, CA, USA; 10 Department of Biomathematics, David Geffen School of Medicine, University of California Los Angeles, Los Angeles, CA 90095, USA; 11 Department of Biostatistics, Fielding School of Public Health, University of California Los Angeles, Los Angeles, CA 90095, USA; 12 Department of Human Genetics, David Geffen School of Medicine, University of California Los Angeles, Los Angeles, CA 90095, USA; 13 Spatial Epidemiology Lab (SpELL), Université Libre de Bruxelles, CP160/12, 50 av. FD Roosevelt, 1050 Bruxelles, Belgium

**Keywords:** COVID-19, SARS-CoV-2, Europe, second wave, phylogeography, international mobility

## Abstract

Following the first wave of SARS-CoV-2 infections in spring 2020, Europe experienced a resurgence of the virus starting late summer that was deadlier and more difficult to contain. Relaxed intervention measures and summer travel have been implicated as drivers of the second wave. Here, we build a phylogeographic model to evaluate how newly introduced lineages, as opposed to the rekindling of persistent lineages, contributed to the COVID-19 resurgence in Europe. We inform this model using genomic, mobility and epidemiological data from 10 West European countries and estimate that in many countries more than 50% of the lineages circulating in late summer resulted from new introductions since June 15^th^. The success in onwards transmission of these lineages is predicted by SARS-CoV-2 incidence during this period. Relatively early introductions from Spain into the United Kingdom contributed to the successful spread of the 20A.EU1/B.1.177 variant. The pervasive spread of variants that have not been associated with an advantage in transmissibility highlights the threat of novel variants of concern that emerged more recently and have been disseminated by holiday travel. Our findings indicate that more effective and coordinated measures are required to contain spread through cross-border travel.

## Introduction

Upon successfully curbing transmission in spring 2020, many European countries witnessed a resurgence in COVID-19 cases in late summer. The number of COVID-19 infections increased rapidly, and by the end of October, it was clear that the continent was deep into a second epidemic wave. In England for example, the number of infections was doubling every nine days and the reproduction number was estimated to have risen to 1.6^1^. By early December, it was clear that the second wave had become deadlier than the first as the number of Europeans that died of COVID-19 in November had surpassed the total number in April ^2^. This forced governments to reimpose new lockdowns and social restrictions in an effort to contain the second wave. While these measures have again reduced infection rates across Europe ^3^, this may only be temporary. Different countries have witnessed a stabilization at relatively high levels or even a new surge in infections. In the United Kingdom (UK), the surge can be largely attributed to the rapid spread of a new variant (B.1.1.7, Variant of Concern 202012/01 or 20I/501Y.V1 ^4^), which appears to be more transmissible across all age groups and therefore more challenging to contain^5^.

Already early in the pandemic, experts warned about secondary waves and modelling studies informed by the seasonal variation of endemic coronaviruses predicted a larger winter peak in the Northern hemisphere ^6^. By mid April, the European Commission constructed a roadmap to lifting coronavirus containment measures ^7^, recommending a cautious and coordinated manner to revive social and economic activities. However, the early start of the devastating second wave demonstrated that, in practice, there was insufficient adherence to these measured recommendations. Cross-border travel, and mass tourism in particular, has been implicated as a major instigator of the second wave. In Belgium for example, which suffered a harsh spring wave and witnessed one of the earliest incidence rises in summer, millions were returning from holidays without testing or enforced quarantine demands. Genomic surveillance demonstrated that a new variant (lineage B.1.177 ^8^, 20A.EU1 [nextstrain.org]) that emerged in Spain in early summer has spread to multiple locations in Europe ^9^. While this variant quickly grew into the dominant circulating SARS-CoV-2 strain in different countries (e.g. the UK and the Netherlands) and illustrated intensive transmission dynamics across countries, it did not appear to be associated with a higher intrinsic transmissibility^9^.

Although it appears clear that travel had a significant impact on the second wave in Europe, it remains challenging to assess how it may have restructured and reignited the epidemic in the different European countries. Even without resuming travel, relaxing containment measures when low-level transmission is ongoing risks the proliferation of locally circulating strains. Hodcroft et al. (2020) demonstrated that genomic analyses provide important insights into the spread of new variants underlying the resurgence dynamics. Specifically, the authors documented how the spread of B1.177/20A.EU1 has impacted the genetic make-up of SARS-CoV-2 in European countries in different ways. Phylodynamic analyses may provide further detail on the relative importance of persistence versus the introduction of new lineages, but such analyses are complicated for SARS-CoV-2 for different reasons. Phylogenetic reconstructions may be poorly resolved due to the relatively limited substitutions accumulating in SARS-CoV-2 over time. This is further confounded by the degree of mixing that can be expected from unrestricted travel prior to the lockdowns in spring 2020. Here, we perform a phylodynamic analysis to evaluate the relative importance of persistence versus new introductions in causing the resurgence in different European countries. To maximize the resolution of our reconstructions, we incorporate epidemiological data and measures of human mobility across countries. Using this approach, we uncover the degree to which introductions over the summer period differentially contributed to the second wave in various European countries.

## Results

### Mobility data predicts phylogeographic patterns of SARS-CoV-2 spread

**Extended Data Figure 1.**
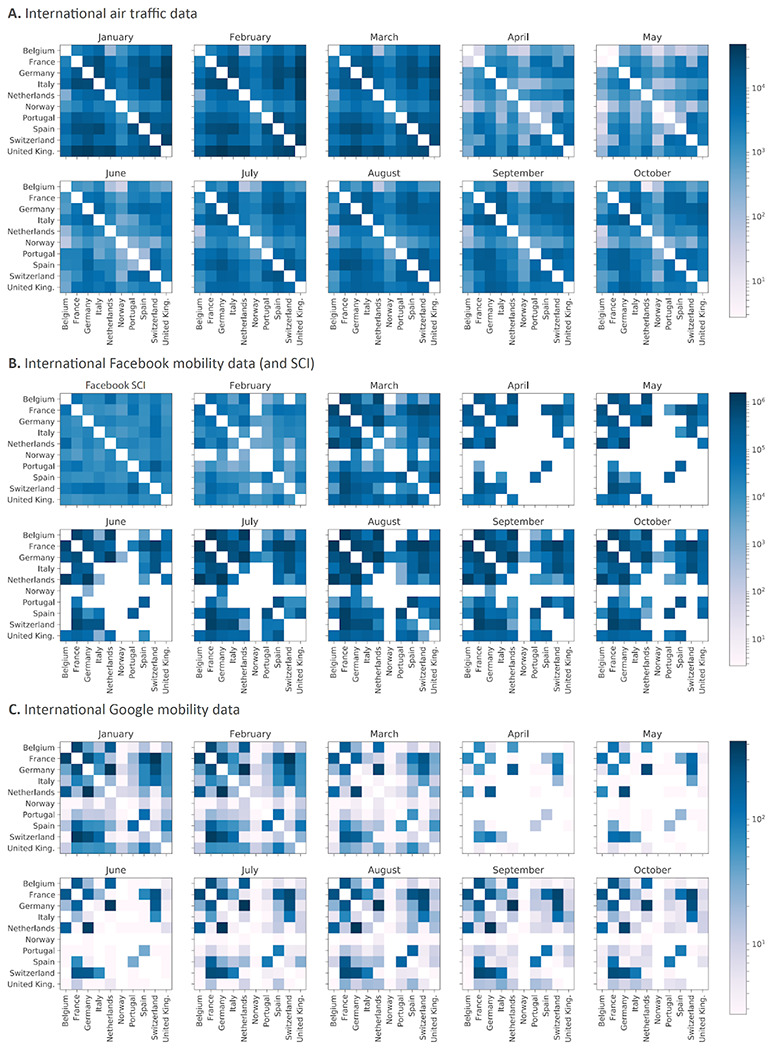
Monthly international mobility data matrices: international air traffic data, international Facebook mobility data, and international mobility data. For Facebook data, we also report the single social connectedness index matrix (SCI, **B**).

We analyzed SARS-CoV-2 B.1 (20A) genomes from 10 European countries for which a minimal number of genomes from the second wave were already available on November 3rd, 2020. By subsampling relative to total case counts, we first compiled a data set of close to 3,000 genomes sampled up to October 20th, 2020, and subsequently updated this data set on January 5th, 2021, to close to 4,000 genomes sampled up to October 30th, 2020 (cfr. Methods, Extended Data Table 1). Due to relatively low sequence diversity, phylogenetic reconstructions of SARS-CoV-2 may be poorly resolved ^10^. In order to achieve maximum resolution, we constructed a Bayesian time-measured phylogeographic model that integrates mobility and epidemiological data. Using this model, we first tested to what extent viral flow across countries can be predicted by mobility or connectivity measures. Specifically, we considered international air transportation data, the Google COVID-19 Aggregated Mobility Research Dataset (also referred to here as ‘mobility data’ for short), as well as Facebook’s Social Connectedness Index (SCI), as covariates of phylogeographic spread (Extended data Figure 1). The Google mobility data contains anonymized mobility flows aggregated over users who have turned on the Location History setting, which is off by default (cfr. Methods). The Social Connectedness Index reflects the structure of social networks and has been suggested to correlate with the geographic spread of COVID-19 ^11^. To help inform the phylogenetic coalescent time distribution, we parameterized the viral population size trajectories through time as a function of incidence data for the 10 countries under investigation.

**Extended Data Figure 2.**
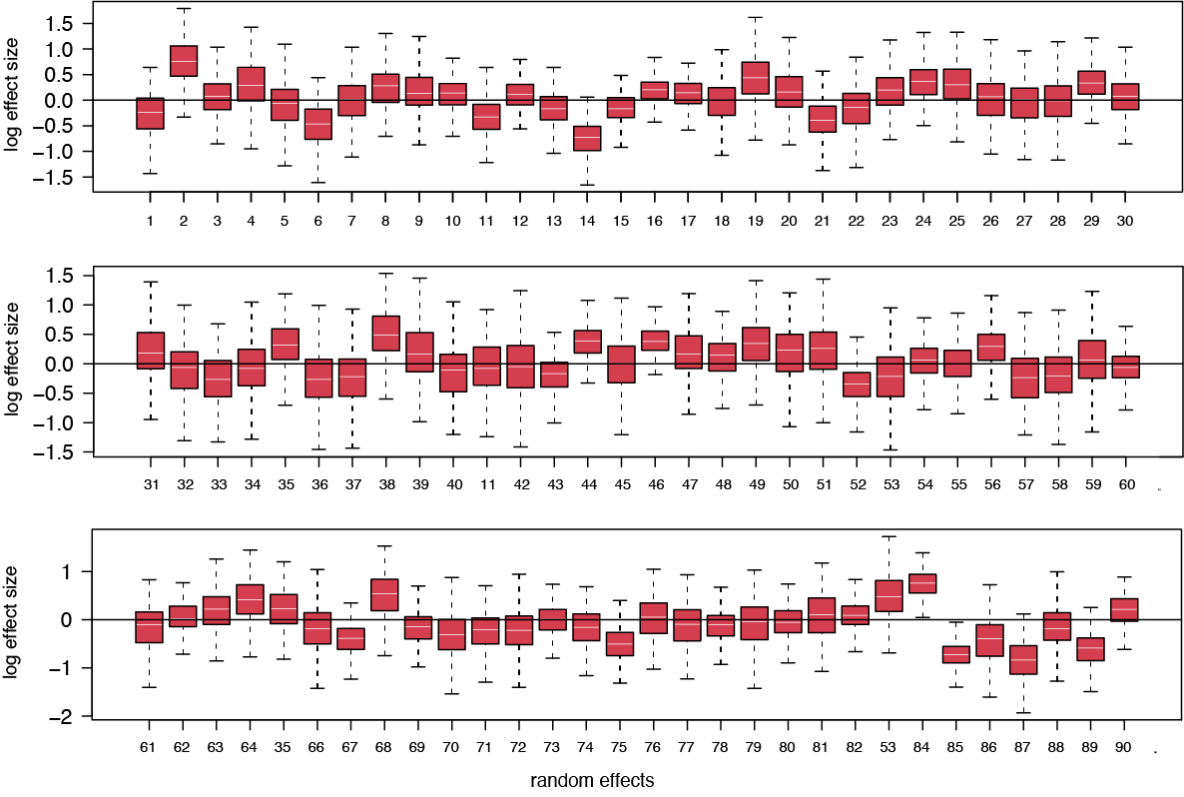
Posterior summary of the GLM random effects. The posterior distribution for each random effect in log space is summarized as an error bar plot. The mean effect size is represented by a white horizontal line while the whiskers represent the 95% HPD intervals.

**Figure 1.**
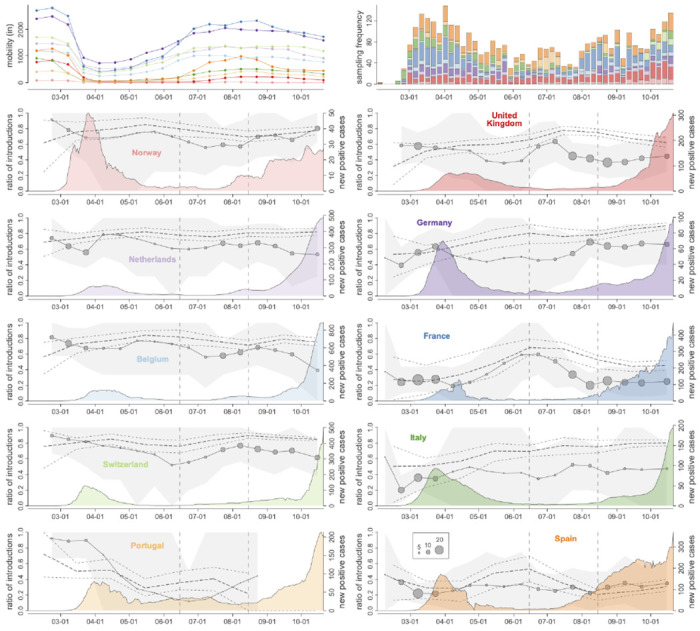
Mobility, genome sampling, case counts and phylogeographic summaries through time for 10 West European countries. The upper left panel summarizes the Google mobility influx by country from the other 10 countries for two-week intervals, while the upper right panel depicts the weekly genome sampling by country used in the phylogeographic analysis. In the remaining panels, we plot for each country the ratio of introductions over the total viral flow from and to that country (for two-week intervals) and a monthly normalized entropy measure summarizing the phylogenetic structure of country-specific transmission chains. The posterior mean ratios of introductions are depicted with circles that have a size proportional to the total number of transitions from and to that country and the grey surface represents the 95% highest posterior density (HPD) intervals. The posterior mean normalized entropies and 95% HPD intervals are depicted by dotted lines. These normalized entropy measures indicate how phylogenetically structured the epidemic is in each country, and ranges from 0 (perfectly structured, e.g a single country-specific cluster) to 1 (unstructured interspersion of country-specific sequences across the entire SARS-CoV-2 phylogeny). The introduction ratios and normalized entropy measures are superimposed over the number of COVID-19 cases reported for each country through time (coloured density plot). The two vertical dashed lines represent the summer time interval (June 15 and August 15, 2020) for which we subsequently evaluate introductions versus persistence (Figure 2).

Using a time-homogeneous model of spatial diffusion, we estimate a maximum inclusion probability and positive log effect size for mobility data whereas air transportation data and SCI offer no predictive value (Table 1). We also estimate a significantly positive association between viral population size change through time and COVID-19 incidence (Table 1). We further confirm the support for the mobility covariate in a time-inhomogeneous spatial model that incorporates monthly mobility measures, with either constant or time-variable inclusion probabilities (Table 1). The fact that mobility data encompassing both air and land-based transport are required to explain COVID-19 spread highlights the need to consider both types of transport in containment strategies. Having associated mobility with phylogeographic dispersal, we focus on this predictor and include its bi-weekly variation in subsequent reconstructions. In addition to parameterizing the relative rates of spread between countries according to this covariate, we extend our time-inhomogeneous approach to also model bi-weekly variation in the overall rate of spread between countries as a function of mobility measures (Fig. 1). This approach estimates a positive association between the overall rate of spatial spread and mobility data (Table 1). Finally, we add time-homogeneous random effects to the phylogeographic transition rates parameterized according to mobility data across epochs in order to account for potential consistent biases in the ability of mobility to predict phylogeographic spread. While posterior mean estimates for the random effects vary, only very few indicate that individual phylogeographic transition rates significantly deviate from the mobility data (Extended Data Figure 2).

### A high degree of genetic mixing and dynamic cross-country transmission through time

**Extended Data Figure 3.**
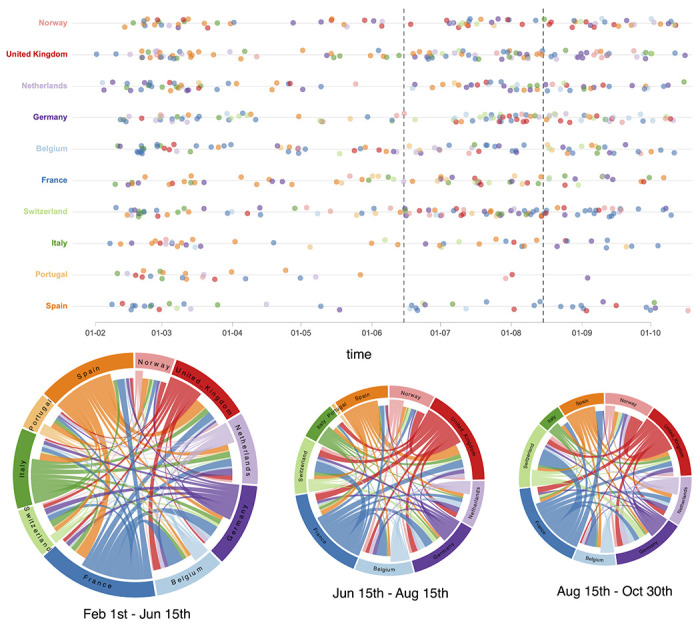
Estimated introductions through time in the 10 European countries and circular migration flow plots summarizing the estimated transitions between the countries for different time intervals throughout the SARS-CoV-2 evolutionary history. The introductions through time serve as an illustration and are based on the Markov jump history in the MCC tree. We note that the posterior distribution of trees is accompanied with considerable uncertainty about the location of origin, destination and timing of the transitions, which is difficult to appropriately visualize. The circular migration flow plots are based on the posterior expectations of the Markov jumps. The size of the plots reflects the total number of transitions for each period. In these plots, migration flow out of a particular location starts close to the outer ring for that origin location whereas migration flow into a particular location ends more distant from the outer ring for that destination location.

We use our probabilistic model of spatial spread informed by genomic data, mobility and epidemiological data to characterize the dynamics of spread throughout the epidemic in Europe. We first focus on the ratio of introductions over the total viral flow in and out of each country over time and the genetic structure of country-specific transmission chains (Fig. 1). For the latter, we use a normalized entropy measure that quantifies the degree of phylogenetic interspersion of country-specific transmission chains in the SARS-CoV-2 phylogeny (cfr. Methods). Although estimates for individual dispersal between pairs of countries can also be obtained (Extended Data Figure 3), we remain cautious in interpreting these as direct pathways of spread because the genome sampling only covers a restricted set of European countries. The mobility to/from each country within our 10-country sample covers between 64% and 96% of the mobility to/from all countries within Europe (Extended Data Table 2), except for Norway (27%), for which other Scandinavian countries account for considerable mobility connections (61%), and the UK (49%), for which Ireland accounts for a large fraction of mobility connections (38%).

According to the proportion of introductions, we estimate more viral import than export events for Norway, the Netherlands, Belgium and Switzerland throughout most of the time period under investigation. According to the estimated phylogenetic entropy, these countries also experienced many independent transmission chains from the beginning of the epidemic spread in Europe. This is consistent with country-specific studies; in Belgium for example, about 331 individual introductions were estimated in the ancestry of a limited sample of 740 genomes^12^. For Portugal, we also estimate higher proportions of introductions early in the first wave but with a subsequent decline to predominantly export events. France, Italy and Spain on the other hand are characterized by a relatively high viral export during the first wave. The proportion of introductions remains relatively low for Italy and Spain following the first wave, while in France these proportions are high from mid June till the end of July. The absolute number of transitions in our sample are however low during this time period. These countries also have comparatively lower entropy values early in the epidemic, with an increase for France by the start of summer and a more gradual increase over time for Italy. In Spain however, the genetic complexity of SARS-CoV-2 transmission chains remains limited. In the UK and Germany, the viral flow in and out of the country is initially relatively balanced. A recent large-scale genomic analysis in the UK indicates that this can imply very high absolute numbers of cross-country transmissions as more than 2,800 independent introduction events were identified from the analysis of 26,181 genomes ^13^. Although our sample is limited compared to this analysis, our reconstructions also recover major influx from Spain, France and Italy during the first wave in the United Kingdom (Extended Data Figure 3). We estimate the highest proportion of introductions for the United Kingdom during the first half of July, indicating an important viral import relative to export around this time. The phylogenetic entropy also peaks around this time. In Germany, the proportions peak somewhat later in summer with a concomitant rise in phylogenetic entropy. We subsequently focus on the time period between the two waves to determine the role of introductions versus persistence in seeding the second wave.

### A high proportion of summer introductions is modulated by local incidence

**Extended Data Figure 4.**
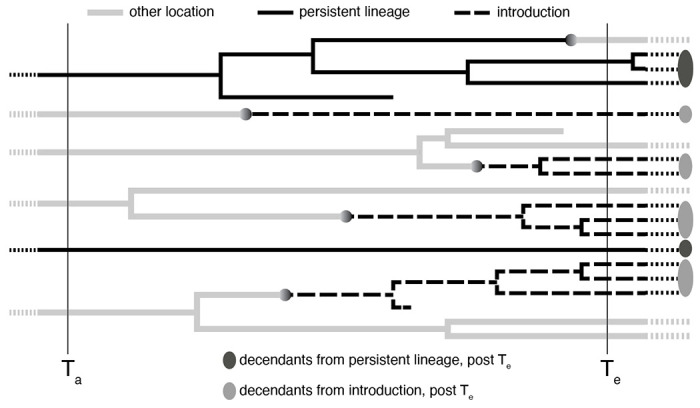
Conceptual representation of persistent lineages and introductions during the time interval delineated by the evaluation time (T_e_) and the ancestral time (T_a_). At T_e_, we evaluate how many lineages are circulating in the location of interest, in this case 12 (lineages in other locations are represented by thick grey branches). We subsequently identify whether these lineages maintained this location up to T_a_ in their ancestry or whether they result from an introduction event in the time interval of interest. By determining whether other lineages circulating in the location of interest at T_e_ are descendants of the same persistent lineage or whether they share an introduction event, we identify the unique persistent lineages or introductions, in this case 2 and 4 respectively. In addition to the proportion of unique introductions (4/6), we also summarize the proportion of their descendants at T_e_ (9/(9+3) in this case) and the proportion of their descendants in terms of sampled tips after T_e_. Those tips are not shown here but conceptually represented for both introductions and persistent lineages by ovals.

**Figure 2.**
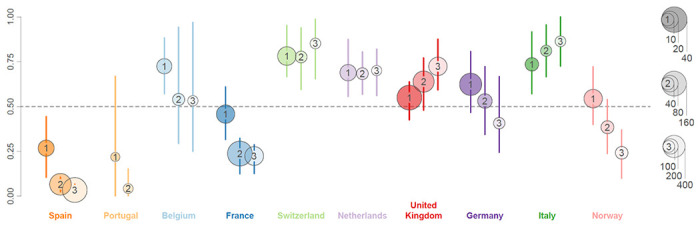
Posterior estimates for relative importance of lineage introduction events among West European countries. For each country, we report three summaries (posterior mean and 95% HPD intervals): (1) the ratio of unique introductions over the total number of unique persisting lineages and unique introductions between June 15 and August 15, 2020, (2) the ratio of descendant lineages from these unique introduction events over the total number of descendants circulating on August 15, 2020, and (3) the ratio of descendant taxa from these unique introductions over the total number of descendant taxa sampled after August 15, 2020 (cfr. Extended Data Figure 4). The dot sizes are proportional to: (1) the total number of unique lineage introductions identified between June 15 and August 15, 2020, (2) the total number of lineages inferred on August 15, 2020, and (3) the total number of descendant sequences after August 15, 2020. The third ratio is not included for Portugal due to insufficient sequences sampled after August 15, 2020.

To assess the impact of summer travel on the second wave in the different countries, we use our genomic-mobility reconstruction to estimate both the number of lineages persisting in each country and the number of newly introduced lineages, and how these proliferated early in the second wave. We focus on a two-month time period between June 15^th^, on which many EU and Schengen-area countries opened their borders to other countries, and August 15^th^, before which the majority of holiday return travel is expected for many countries. We identify the number of lineages circulating in each country on August 15^th^, and determine whether they result from a lineage that persisted since June 15^th^ or from a unique introduction after this date, so independent of the number of descendants for this lineage on August 15^th^ (Extended Data Figure 4). In Figure 2, we plot i) the ratio of these unique introductions over the total unique lineages (unique introductions and persisting lineages), ii) the proportion of descendant lineages on August 15^th^ that resulted from the unique introductions over the total descendants circulating on this date and iii) the proportion of descendant tips (sampled genomes) after August 15^th^ that resulted from the unique introductions over the total number of descendant tips (cfr. Methods and Extended Data Figure 4). The latter two proportions provide an assessment of how the unique introductions and persisting lineages evolved up to, and after, August 15^th^. We estimate a posterior mean proportion of unique introductions that is close to or higher than 0.5 except for Spain and Portugal. This indicates that by August 15^th^ a relatively large fraction of circulating lineages in each country resulted from new introductions over the summer. Because we compare introductions and persistence relatively early (and irrespective of their number of descendants), and because the major variant involved appears not to be associated with increased transmissibility ^9^, we consider the newly introduced lineages to be additional transmission chains that did not necessarily have to compete with persistent transmission chains for susceptibles. However, the two proportions of descendants from these introductions on August 15^th^ and after this date measure their relative success compared to persisting lineages, indicating considerable variation in onwards transmission. The country estimates are ordered according to decreasing average incidence during the June 15 - August 15 time period, suggesting that incidence may shape the outcome of the introductions. In countries that experienced relatively high summer incidence, e.g. Spain, Portugal, Belgium and France, the introductions lead to comparatively fewer descendants on August 15^th^ or after. Although the introductions in Norway also lead to fewer descendants despite the country having the lowest incidence, we find a significant overall association between incidence and the difference in the logit proportion of unique introductions and the logit proportion of their descendants on August 15^th^ (*p* = 0.01). Norway may to some extent be an outlier because persistent lineages in this country could in fact be introductions from other Scandinavian countries that are not represented in our genome sample.

**Extended Data Figure 5.**
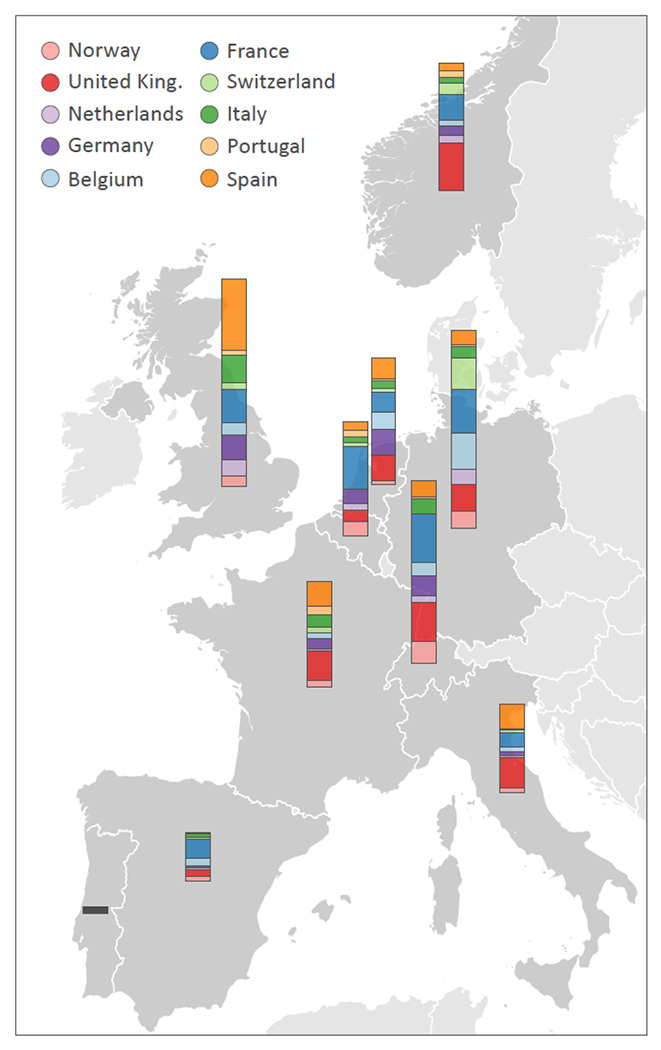
Estimated geographic origin of viral influx over the summer n(June 15th - August 15th, 2020) in each country. Each barplot summarizes the posterior Markov jump estimates into a specific country.

**Extended Data Figure 6.**
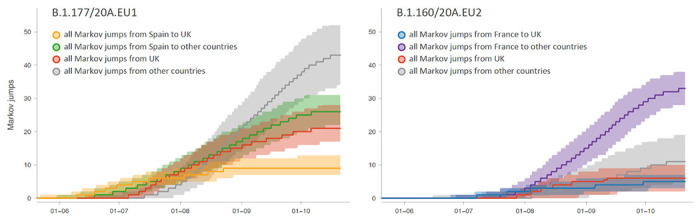
Phylogeographic transitions for lineages B1.1777/20A.EU1 and B1.160/20A.EU2. Cumulative phylogeographic transitions are summarized as posterior mean estimates with 95% HPD intervals over time for 4 types of Markov jumps. For B1.1777/20A.EU1: i) from Spain to the United Kingdom (UK), ii) from Spain to other countries, iil) from the UK, and iv) from other countries; For B1.160/20A.EU2: i) from France to the UK, ii) from France toother countries, iil) from the UK, and iv) from other countries.

**Figure 3.**
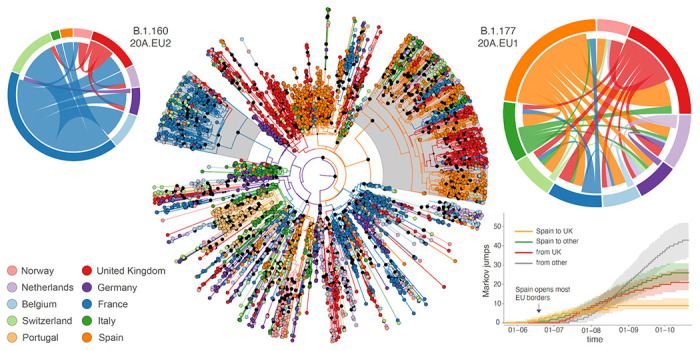
Phylogeographic estimates of SARS-CoV-2 spread in western Europe. The radial tree in the center represents the maximum clade credibility tree summary of the Bayesian inference. Colors correspond to the countries in the legend. Two clades corresponding to B1.1777/20A.EU1 and B1.160/20A.EU2 are highlighted in grey. The circular migration flow plots for these variants are based on the posterior expectations of the Markov jumps. In these plots, migration flow out of a particular location starts close to the outer ring for that origin location whereas migration flow into a particular location ends more distant from the outer ring for that destination location. For B1.1777/20A.EU1, we summarize phylogeographic transitions as mean estimates with 95% HPD intervals over time for 4 types of Markov jumps: i) from Spain to the United Kingdom, ii) from Spain to other countries, iil) from the United Kingdom, and iv) from other countries.

Our estimates show a marked increase in descendants from introductions in the United Kingdom (Figure 2), with a considerable fraction of introductions originating from Spain (Extended Data Figure 5) likely reflecting the spread of B.1.177/20A.EU1 that rapidly became the most dominant strain in the United Kingdom ^9^. Our analysis captures the expansion of this variant as well as that of B.1.160/20A.EU2, which together account for more than 25% of the genomes in our sample. While Spain is indeed inferred to be the origin of B.1.177/20A.EU1, the United Kingdom also considerably contributed to its spread (Figure 3). The earliest introduction from Spain to the United Kingdom is estimated around the time Spain opened most EU borders (June 21^st^, Figure 3). While introductions from Spain to other countries soon followed, we estimate a similar rate and amount of spread from the United Kingdom to other countries before these other countries also disseminate the virus. Whereas our sample remains limited, it illustrates a dynamic pattern of spread and the importance of the early establishment of B.1.177/20A.EU1 in the United Kingdom that served as an important secondary center of dissemination. While the United Kingdom is also to some extent involved in the spread of B1.160/20A.EU2, this variant has been largely disseminated from France. The simple fact this variant expanded later in France and subsequently started to spread later than B.1.177/20A.EU1 (Extended Data Figure 6) may explain why the latter spread more successfully.

## Discussion

In this study, we gain insight into the dynamics of SARS-CoV-2 spread through phylogeographic analyses, specifically focusing on the impact of European travel during the summer of 2020. Because such analyses may suffer from a lack of resolution offered by SARS-CoV-2 genomic data, we integrate epidemiological and mobility data to help shape the phylodynamic process. Our model supports mobility data, including both air and land transportation, as a predictor of viral flow between countries. The resulting reconstructions show that the composition of lineages circulating towards the end of the summer was to a significant extent shaped by introductions in most of the European countries. Interestingly, the relative success of onwards transmission of the introduced lineages appears to be shaped by the average summer COVID-19 incidence. In countries that maintained a relatively high incidence, e.g. Spain, Portugal, Belgium and France, these introductions resulted in comparably less onwards transmission over relatively short-term than for the lineages that persisted in the countries over summer.

As documented by Hodcroft et al. (2020) ^9^, SARS-CoV-2 spread during summer in Europe involved to a large extent the B.1.177/20A.EU1 variant. This variant became the dominant strain relatively early in the UK ^9^, facilitating its further spread over the second half of summer. A limited sample offers only a limited view of the transmission history of this variant; in depth analyses of close to 20,000 20A.EU1 genomes indicate hundreds of introductions to countries across Europe ^9^. Importantly however, there is no evidence of increased transmissibility of this variant and its success is currently attributed to repeated introductions upon resuming travel with insufficient effective containment strategies^9^.

Our results should be interpreted in light of several important limitations. Although about 4,000 genomes constitute a large dataset for Bayesian phylodynamic inference, it remains a limited sample to infer detailed COVID-19 transmission dynamics. In addition, the genome data do not cover all Western European countries, implying that we are missing transmission events that involve unsampled countries. This may be particularly important for Norway for example, which according to our mobility data, is largely connected to other Scandinavian countries. Also, the mobility data are subject to limitations as these may not be representative for the population as whole and their representativeness may vary by location.

The significant mixing and rapid spread to high frequencies of variants that are not associated with higher transmissibility underscores the risk for pervasive spread of variants that are more transmissible, like B.1.1.7 (Variant of Concern 202012/01,20I/501Y.V1) in the United Kingdom, or could be more transmissible, like B.1.351 (20H/501Y.V2) in South Africa and variant P1 in Brazil. Different from the rise in frequency of 20A.EU1 due to repeated introductions in the UK, B.1.1.7 appears to have emerged from within the UK. Preliminary findings demonstrate a consistent repeated pattern of faster epidemic growth of B.1.1.7 and provide evidence for a significant transmission advantage over prior lineages ^5^. The consequences for control of COVID-19 have become clear for the United Kingdom. As of January 19,2021, approximately 16,800 B.1.1.7 cases have been identified in the UK and approximately 2,000 cases have been identified in 60 other countries ^14^. More intense genomic surveillance is needed across Europe to track this variant, and control of COVID-19 in general would benefit from strengthened coordination. Disruptive border closures (e.g. between France and UK, on December 21-22, 2020) can only serve as a short-term emergency measure to put into place better strategies to prevent between-country transmission. No matter how much more transmisible SARS-CoV-2 variants may be, quarantining, testing and social distancing remain effective when adhered to and they will be required for some time even as vaccination programs are being rolled out.

## Methods

### Sequence data and subsampling

We used a two-step genome data collection procedure. We first evaluated the available genomes from European countries in GISAID ^15^ on November 3,2020. We selected genomes from Belgium, France, Germany, Italy, Netherlands, Norway, Portugal, Spain, Switzerland and the United Kingdom primarily based on the availability of genome data from both the first and second wave at that time but also because of their high ratio of genomes to positive cases. Portugal represented an exception because data for this country were limited to the first wave at that time, but we included genomes from Portugal because of its potential importance as a summer travel location.

We aligned the genomes from each country using MAFFT v7.453 ^16^ and trimmed the 5′ and 3′ ends and only retained unique sequences from each location. To further mitigate the disparities in sampling, we subsampled each country proportionally to the cumulative number of cases on October 21^st^ (the most recently sampled sequence at the time) by setting an arbitrary threshold of 6.5 sequences per 10,000 cases, with a minimum number of 100 sequences per country. To maximize the temporal and spatial coverage in each country, we binned genomes by epi-week and sampled as evenly as possible, sampling from a different region within the country when available. Only sequences from the B.1 lineage with the D614G mutation and exact sampling dates were selected for the analyses. From the final aligned sequence set, we removed 12 potential outliers, based on a root-to-tip regression on TempEst v1.5.3^17^ on a maximum-likelihood tree inferred with IQTREE v2.0.3^18^, yielding a data set of 2,909 genomes (Extended Data Table 1).

Because of the nature of genome sequence accumulation, fewer recently sampled genomes were available for most countries on November 3rd (relative to the case counts at this time). Because our primary goal was to assess the persistence and introduction of lineages leading up to the second wave, we sought to augment our data set with more recent genomes, having already performed analyses on the initial data set. In the section on Bayesian evolutionary reconstructions, we outline how we update these analyses accordingly. On January 5, 2021, we updated our dataset by adding over 1,000 non-identical sequences collected between August 1^st^ and October 31^st^. For Portugal, we extended this period back to June 22^nd^ (the most recent sampling date for the previous Portuguese selection). We downloaded all new B.1 sequences with the D614G mutation collected during the selected time period from GISAID and performed the following subsampling. The number of genomes to add by country was obtained by raising the threshold ratio of sequences/cases to 8.5 and increasing the minimum number of sequences to 200. To bias the temporal coverage towards more recent samples, the genomes from each country were binned by week and sampled such that the number of sequences added by week was proportional to an exponential function of the form *e*^*t*/4^, where *t*=0 represents August 1^st^ and *t*=13 is October 31^st^. For Portugal, we did not use this preferential sampling as we needed to include close to all available genomes to raise the number of genomes to 200. The sampled sequences were then deduplicated and outliers were removed as described in the previous section. With the additional selection of 1,050 genomes, we arrived at a data set of 3,959 genomes (Extended Data Table 1).

### Mobility data

We analysed four different mobility/connectivity metrics: air traffic flows, a social connectedness index provided by Facebook, as well as aggregate Google and Facebook international mobility data. Air traffic flow data were obtained from the International Air Transport Association (http://www.iata.org) and based on the number of origin-destination tickets while also taking into account connections at intermediate airports (Gilbert et al. 2020). We used monthly air traffic data between the 10 western European countries under investigation for the time period between January 2020 and October 2020. The social connectedness index (SCI) is an anonymized snapshot of active Facebook users and their friendship networks to measure the intensity of social connectedness between countries (https://data.humdata.org/). In practice, the SCI measures the relative probability of a Facebook friendship link between two users of the application in different countries. We used the SCI calculated for the 10 Western european countries as of August 2020.

The Google COVID-19 Aggregated Mobility Research Dataset contains anonymized mobility flows aggregated over users who have turned on the Location History setting, which is off by default. To produce this dataset, machine learning is applied to logs data to automatically segment it into semantic trips ^19^. To provide strong privacy guarantees, all trips were anonymized and aggregated using a differentially private mechanism ^20^ to aggregate flows over time (see https://policies.google.com/technologies/anonymization). This research was done on the resulting heavily aggregated and differentially private data. No individual user data was ever manually inspected, only heavily aggregated flows of large populations were handled. All anonymized trips were processed in aggregate to extract their origin and destination location and time. For example, if users traveled from location *a* to location *b* within time interval *t*, the corresponding cell (*a, b, t*) in the tensor would be *n* ± *η*, where *η* is Laplacian noise. The automated Laplace mechanism adds random noise drawn from a zero-mean Laplace distribution and yields (*ϵ, δ*)-differential privacy guarantee of *ϵ* = 0.66 and *δ* = 2.1 × 10-29 per metric. The parameter *ϵ* controls the noise intensity in terms of its variance, while *δ* represents the deviation from pure *ϵ*-privacy. The closer they are to zero, the stronger the privacy guarantees. We used aggregated mobility flows between the 10 western European countries and summarized by two-week or monthly time periods between January 2020 and October 2020.

**Extended Data Figure 7.**
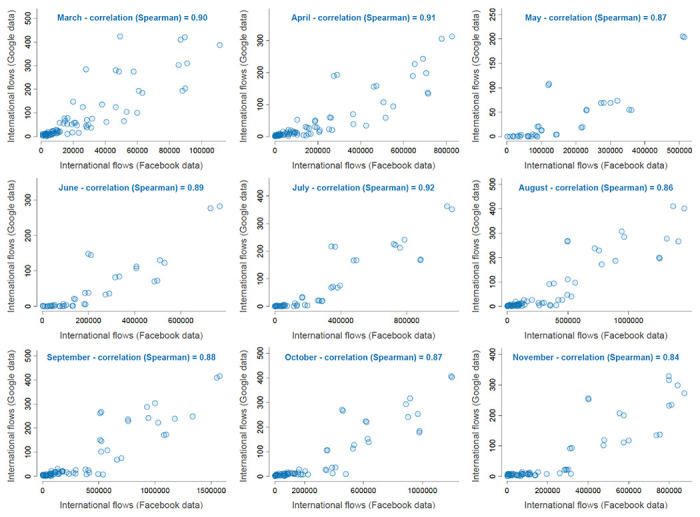
Comparison between Google and Facebook aggregate international mobility data. We summarize monthly correlations using scatter plots and Spearman’s rank correlation. Each dot in the scatter plots corresponds to a specific pair of European countries considered in our study.

Finally, we also considered international mobility data from Facebook mobility data as an alternative to Google mobility data. These data are based on numbers of Facebook users moving over large distances, like air or train travel. Counts of international travel patterns are updated daily based only on users who have opted into sharing precise location data from their device with the Facebook mobile app through location services. Also in this case, we used aggregated mobility flows between the 10 western European countries summarized by month between January 2020 and October 2020. Because international aggregate mobility data obtained from Google and Facebook are highly correlated (monthly Spearman correlation ranging from 0.84 to 0.92; Extended Figure 7), we only included the Google aggregate mobility data as a covariate in the phylogeographic analyses.

### Bayesian evolutionary reconstructions

#### - Joint sequence-trait inference with a time-homogeneous GLM diffusion model

**Extended Data Figure 8.**
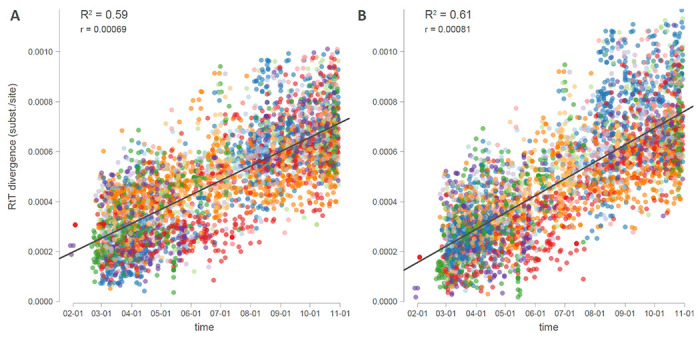
Root-to-tip divergence as a function of sampling time for the 3959 genome data set with a different rooting of the same maximum likelihood tree. **A.** Tree rooted according to the best-fitting root under the heuristic residual mean squared criterio. **B.** Tree rooted along the branch leading to the cluster of 3 Bavarian genomes that resulted from an independent introduction into Europe.

We performed Bayesian evolutionary reconstruction of timed phylogeographic history using BEAST 1.10 ^21^ incorporating genome sequences, their country and date of sampling, epidemiological and mobility/connectivity data. Because of the relatively low degree of resolution offered by the sequence data, our full probabilistic model specification focuses on i) relatively simple model specifications and ii) informing parameters by additional non-genetic data sources. We modeled sequence evolution using an HKY85 nucleotide substitution model with gamma-distributed rate variation among sites and a strict molecular clock model. Our genome set includes three genomes from an early outbreak in Bavaria, which was caused by an independent introduction from China ^22,23^. We therefore constrained these genomes as an outgroup in the analysis, which according to root-to-tip regression plots as a function of sampling time resulted in a better correlation coefficient/R-squared compared to the best-fitting root under the heuristic mean residual squared criterion (Extended Figure 8)^17^.

As a coalescent tree prior, we modeled the effective population size trajectory as a piecewise constant function that changes values at pre-specified times ^following 24^ with log population sizes modelled as a deterministic function of log COVID-19 case counts ^following 25^. This reduces the nonparametric skygrid parameterization to a generalized linear model (GLM) formulation with an estimable regression intercept and coefficient. Specifically, we used two-week intervals and specified as a covariate the total case counts over these time intervals for the 10 countries of sampling. The earliest interval with non-zero cases counts was from 2020-01-14 to 2020-01-28; before 2020-01-14, the log-transformed and standardized case count covariate was set to the equivalent of 1 case. Case count data were obtained from https://www.ecdc.europa.eu/en/covid-19/data.

Similar to sequence evolution, we modelled the process of transitioning through discrete location states (countries of sampling) according to a continuous-time Markov chain (CTMC) ^26^. We employed a parameterization that models the log transition rates as a log linear function of mobility/connectivity covariates ^27^. As covariates we considered Facebook’s SCI, air transportation data and mobility data. For the two time-variable mobility measures, we used the average of the log-transformed and standardized monthly mobility measures as a single covariate in our time-homogeneous phylogeographic GLM model. In addition to estimating the contribution (effect size) of each covariate in this GLM, we also estimated their inclusion probabilities through a spike-and-slab procedure.

We performed inference under the full model specification using Markov chain Monte Carlo (MCMC) sampling and used the BEAGLE library v3^28^ to increase computational performance. We specified standard transition kernels on all parameters, except for the regression coefficients of the piecewise-constant coalescent GLM model. For these parameters, we implemented new Hamiltonian Monte Carlo (HMC) transition kernels to improve sampling efficiency. These kernels use principles from Hamiltonian dynamics and their approximate energy conserving properties to reduce correlation between successive sampled states, but require computation of the gradient of the model log-posterior with respect to the parameters of interest, in addition to efficient evaluation of the log-posterior that BEAGLE provides. To accomplish this, we extended our previous analytic derivation of the gradient of the log-density from the skygrid coalescent model with respect to the log-population-sizes ^29^ to now be with respect to the regression coefficients using the chain rule and their regression design matrix.

Due to the data set size, MCMC burn-in takes up considerable computational time. We therefore iterated through a series BEAST inferences, initially only considering sequence evolution and subsequently adding the location data, to arrive at a tree distribution from which trees were taken as starting trees in our final analyses. The latter was composed of multiple independent MCMC runs that were run sufficiently long to ensure that their combined posterior samples achieved effective sample sizes (ESSs) larger than 100 for all continuous parameters.

#### - Data augmentation through online BEAST

As we updated our dataset following initial analyses of the 2,909 genome collection using the approach discussed in the previous subsection, we sought to capitalize on these efforts to limit the burn-in for subsequent analyses of the 3,959 dataset. Specifically, we adopted the distance-based procedure to insert new taxa into a time-measured phylogenetic tree sample as implemented in the BEAST framework for online inference^30^. We subsequently use the augmented tree as starting tree for the analyses of the updated dataset.

#### - Time-inhomogeneous reconstructions

To accommodate the time-variability of the mobility measures, we constructed epoch model extensions of the discrete phylogeography approach that allow specifying arbitrary intervals over the evolutionary history and associating them with different model parameterizations ^31^. As a complement to testing covariates of spatial diffusion using a time-homogeneous model, we used the epoch extension to specify monthly intervals allowing us to incorporate monthly mobility matrices (air transportation data was only available as monthly numbers), but assuming time-homogeneous effect sizes and inclusion probabilities. Monthly covariates were again log-transformed and standardized after adding a pseudocount to each entry in the monthly matrices.

In addition, we performed another analysis in which we relaxed the constant-through-time inclusion probability of the covariates. In this model specification, each interval is associated with a specific set of indicator variables to represent the inclusion/exclusion of covariates, but we pool information about predictor inclusion across the intervals using hierarchical graph modelling^32^. This approach uses a set of indicator variables to model covariate inclusion at the hierarchical level but allows interval-specific inclusion or predictors to diverge from the hierarchical level with a non-zero probability (with the number of differences modelled as a binomial distribution,^32^), which set to 0.10 in our case. We estimated hierarchical and interval-level inclusion using spike-and-slab.

Finally, we performed an analysis using the time-inhomogeneous model in which the interval-specific transition rates are modelled as a function of the single covariate that is supported by the analyses above leveraging aggregate mobility. We incorporated more variability through time by specifying two-week intervals (similar to the coalescent GLM interval specification). The time-inhomogeneous GLM approach we employ allows modelling relative differences in transition rates, but also the overall rate of migration between countries varies through time and likely more than the relative preferences of migration. Therefore, we further extended this model by incorporating a time-inhomogeneous overall CTMC rate scaler and parameterize it as a log linear function of the total monthly between-country log-transformed and standardized mobility. To generate realisations of the discrete location CTMC process and obtain estimates of the transitions (Markov jumps) between states under this model, we employed posterior inference of the complete Markov jump history through time^27,33^.

While the epoch model allows us to flexibly accommodate time-variable spatial dynamics, it considerably increases the computational burden associated with likelihood evaluations. In order to efficiently draw inference under this model for our large data set, we fit the time-inhomogeneous spatial diffusion process to a set of trees inferred under the time-homogeneous GLM diffusion model described above. Although likelihood evaluations remain computationally expensive, even with the speed-up offered by GPU computation with BEAGLE, eliminating simultaneous tree estimation tremendously reduces parameter-space, requiring only modest MCMC chain lengths to adequately explore it.

#### - Posterior Summaries

We assessed MCMC mixing (e.g. using ESSs) and summarized continuous parameter estimates using Tracer v1.7.1 ^34^. Credible intervals were computed as 95% HPD intervals. Trees were visualized using FieTree v1.4.4 (available at https://github.com/rambaut/figtree/releases). In terms of phylogeographic estimates, we mainly focused on i) transitions to each location and from each location (based on Markov jump estimates) instead of pairwise transitions, ii) ratios of these transitions and iii) how these transitions structured transmission chains in individual countries. Transitions to each and from each location avoid drawing conclusions about direct migration between countries, which can be tenuous given the incomplete genomes coverage of Europe, while their ratios avoid using absolute numbers of transitions, which are highly sample-dependent. Phylogeographic inference is limited to reconstructing the transitions in the ancestral history of a sample of sequences, which will only be a small fraction of the actual migration events especially when these events result in insufficient onwards transmission to be captured in our limited sample. In addition, SARS-CoV-2 genome data can be poorly resolved and identical genomes in different locations are consistent with hypotheses that involve both a sparse and a rich number of virus flows between these locations. As the data hold little information to distinguish these hypotheses, we only consider sparse scenario’s by including only unique sequences for each location. A joint inference of sequence evolution and discrete spatial diffusion would err on the side of sparse hypotheses anyway because it will tend to cluster identical sequences that share a location. Despite the general underestimation of spatial dispersal, a phylogeographic inference is still likely to capture the transition events with important onward transmission, and evaluating the importance of such events relative to persistence is a major focus of this study.

We provide three new tree sample tools in the BEAST codebase available at https://github.com/beast-dev/beast-mcmc) to obtain posterior summaries of location transition histories using posterior tree distributions annotated with Markov jumps:

- *TreeMarkovJumpHistoryAnalyzer* allows collecting Markov jumps and their timings from a posterior tree distribution annotated with Markov jumps histories in a .csv file for further analyses.
- *TreeStateTimeSummarizer* decomposes the total tree time into the times associated with contiguous partitions of a tree associated with a particular location state, with the partitions determined by the Markov jumps. An arbitrary lower and upper time boundary can be specified to restrict the summary to a particular time interval in the evolutionary history. We use the time estimates for the separate partitions associated with each state to calculate an entropy measure that summarizes the genetic make-up of country-specific transmission chains. Specifically, we use for each location a normalized Shannon entropy:
  (1) $$-\frac{1}{ln(n)}\sum_{i}^{n}p_{i}ln(p_{i}),$$
  Where *p_i_* is the proportion of time associated with that location for partition *i* of a phylogeographic tree and *n* represents the number of partitions for that location in the tree.
- *PersistenceSummarizer* also uses posterior tree distributions annotated with Markov jumps to summarize the number of lineages at a particular point in time (evaluation time, *T_e_*, cfr. Extended Figure 5), which location states they are associated with, since what time point in the past they have maintained that state and how many sampled descendants they have after time *T_e_* (Extended Figure 5). In addition, it allows identifying how long these lineages have circulated independently prior to *T_e_*, so before sharing common ancestry with other lineages that maintained the same location state. This information allows us to determine how many lineages are circulating at *T_e_* that stem either from a unique persistent lineage (maintaining the same location states) or unique introduction event since a particular time prior to *T_e_*. The association between incidence and the difference in the logit proportion of unique introductions and the logit proportion of their descendants on August 15th was evaluated using *p*-value obtained by a linear regression analysis.

### Data availability

BEAST XML input files are available at https://github.com/phylogeography/SARS-CoV-2_EUR_PHYLOGEOGRAPHY

The SARS-CoV-2 genome data required for running these xmls can be downloaded from https://www.gisaid.org. The Google COVID-19 Aggregated Mobility Research Dataset used for this study is available with permission from Google LLC. The Facebook mobility data can be requested from Facebook (https://dataforgood.fb.com/). COVID-19 incidence data was obtained from https://www.ecdc.europa.eu/en/covid-19/data.

### Code availability

The code for running BEAST analyses is available in the hmc-develop branch of the BEAST codebase available at https://github.com/beast-dev/beast-mcmc. The tools *TreeMarkovJumpHistoryAnalyzer, TreeStateTimeSummarizer and PersistenceSummarizer* are available from the master branch in the same codebase.

## Extended Data

**Extended Data Table 1.** Genome sampling by country, collected on Nov. 3^rd^, 2020, and updated on Jan 5t^h^, 2021.

country	genomes (Nov. 3rd, 2020)	genomes (Jan 5th, 2021)	total
Belgium	183	53	236
France	600	167	767
Germany	246	75	321
Italy	281	75	356
The Netherlands	159	47	206
Norway	100	92	192
Portugal	100	100	200
Spain	647	191	838
Switzerland	100	98	198
The United Kingdom	493	152	645
total	2909	1050	3959

**Extended Data Table 2.** mobility to or from each country within our 10 country sample as the percentage of the total between-country mobility within Europe.

country	Mobility percentage
Belgium	87.2
France	89.5
Germany	63.9
Italy	64.8
The Netherlands	93.2
Norway	27.1
Portugal	94.0
Spain	90.3
Switzerland	84.8
The United Kingdom	48.6

## Supplementary Material

Supplement

## Figures and Tables

**Table 1.** Parameter estimates for the various Bayesian time-measured phylogeographic models applied to the 3,959 genome data set. The coalescent generalized linear model (GLM) parameterizes bi-weekly effective population sizes as a log-linear function of COVID-19 incidence data, with α and β representing the log intercept and log regression coefficient. In the time-inhomogeneous spatial diffusion models, no coalescent prior was used as these models were fitted onto posterior trees inferred from the time-homogeneous model (cfr. Methods). For the spatial GLM model, we report inclusion probability estimates through the expectations of the boolean indicators (δ) associated with each predictor and log conditional effect sizes (the regression coefficient conditional on the predictor being included in the model, β(|δ=1)). SCI = Social Connectedness Index, based on Facebook data. For the model with time-variable inclusion probabilities, we report the parameters at the hierarchical level (δ_h_ and β|δ_h_, cfr. Methods). In the model with a time-variable rate scalar, we parameterize this rate scalar as a log-linear function of the overall between-country mobility, with α and β representing the log intercept and log regression coefficient.

Model		Parameter estimates
Time-homogenous spatial diffusion	coalescent GLM spatial GLM	α = 2.44 [2.35,2.53], β = 1.77 [1.54,1.92] air travel: E[δ] = 0.01, (β|δ=1) = −0.03 [−0.16,0.09] SCI: E[δ] = 0.01, β(|δ=1) = −0.15 [−0.27,−0.02] mobility: E[δ] > 0.99, β(|δ=1) = 0.36 [0.24,0.49]
Time-inhomogeneous spatial diffusion	spatial GLM, constant inclusion probabilities	air travel: E[δ] = 0.05, β(|δ=1) = −0.07 [−0.21,0.07] SCI: E[δ] = 0.16, β|δ=1 = −0.13 [−0.29,0.01] mobility: E[δ] > 0.99, β(|δ=1) = 0.35 [0.22,0.49]
	spatial GLM, time-variable inclusion probabilities	air travel: E[δ_h_] = 0.03, β|(δ_h_=1) = 0.10 [−0.24,0.12] SCI: E[δ_h_] = 0.12, β|δ_h_=1 = −0.14 [−0.35,0.01] mobility: E[δ_h_] = 0.93, β(|δ_h_=1) = 0.39 [0.24,0.57]
	spatial GLM time-variable rate scalar GLM	mobility: β = 0.26 [0.12,0.41] mobility: α = 0.67 [0.54,0.78], β = 0.47 [0.32,0.62]
